# Biomarker Testing Disparities in Metastatic Colorectal Cancer

**DOI:** 10.1001/jamanetworkopen.2024.19142

**Published:** 2024-07-05

**Authors:** Saad Sabbagh, María Herrán, Ali Hijazi, Iktej Singh Jabbal, Mohamed Mohanna, Barbara Dominguez, Mira Itani, Kaylee Sarna, Hong Liang, Zeina Nahleh, Steven D. Wexner, Arun Nagarajan

**Affiliations:** 1 Department of Hematology-Oncology, Maroone Cancer Center, Cleveland Clinic Florida, Weston; 2 Department of Internal Medicine, Advent Health Sebring, Sebring, Florida; 3 Department of Clinical Research, Cleveland Clinic Florida, Weston; 4 Ellen Leifer Shulman and Steven Shulman Digestive Disease Center, Cleveland Clinic Florida, Weston

## Abstract

**Importance:**

Among patients with metastatic colorectal cancer (mCRC), data are limited on disparate biomarker testing and its association with clinical outcomes on a national scale.

**Objective:**

To evaluate the socioeconomic and demographic inequities in microsatellite instability (MSI) and *KRAS* biomarker testing among patients with mCRC and to explore the association of testing with overall survival (OS).

**Design, Setting, and Participants:**

This cohort study, conducted between November 2022 and March 2024, included patients who were diagnosed with mCRC between January 1, 2010, and December 31, 2017. The study obtained data from the National Cancer Database, a hospital-based cancer registry in the US. Patients with mCRC and available information on biomarker testing were included. Patients were classified based on whether they completed or did not complete MSI or *KRAS* tests.

**Exposure:**

Demographic and socioeconomic factors, such as age, race, ethnicity, educational level in area of residence, median household income, insurance type, area of residence, facility type, and facility location were evaluated.

**Main Outcomes and Measures:**

The main outcomes were MSI and *KRAS* testing between the date of diagnosis and the date of first-course therapy. Univariable and multivariable logistic regressions were used to identify the relevant factors in MSI and *KRAS* testing. The OS outcomes were also evaluated.

**Results:**

Among the 41 061 patients included (22 362 males [54.5%]; mean [SD] age, 62.3 [10.1] years; 17.3% identified as Black individuals, 78.0% as White individuals, 4.7% as individuals of other race, with 6.5% Hispanic or 93.5% non-Hispanic ethnicity), 28.8% underwent *KRAS* testing and 43.7% received MSI testing. A significant proportion of patients had Medicare insurance (43.6%), received treatment at a comprehensive community cancer program (40.5%), and lived in an area with lower educational level (51.3%). Factors associated with a lower likelihood of MSI testing included age of 70 to 79 years (relative risk [RR], 0.70; 95% CI, 0.66-0.74; *P* < .001), treatment at a community cancer program (RR, 0.74; 95% CI, 0.70-0.79; *P* < .001), rural residency (RR, 0.80; 95% CI, 0.69-0.92; *P* < .001), lower educational level in area of residence (RR, 0.84; 95% CI, 0.79-0.89; *P* < .001), and treatment at East South Central facilities (RR, 0.67; 95% CI, 0.61-0.73; *P* < .001). Similar patterns were observed for *KRAS* testing. Survival analysis showed modest OS improvement in patients with MSI testing (hazard ratio, 0.93; 95% CI, 0.91-0.96; *P* < .001). The median (IQR) follow-up time for the survival analysis was 13.96 (3.71-29.34) months.

**Conclusions and Relevance:**

This cohort study of patients with mCRC found that older age, community-setting treatment, lower educational level in area of residence, and treatment at East South Central facilities were associated with a reduced likelihood of MSI and *KRAS* testing. Highlighting the sociodemographic-based disparities in biomarker testing can inform the development of strategies that promote equity in cancer care and improve outcomes for underserved populations.

## Key Points

**Question:**

What are the social and demographic factors associated with the receipt of biomarker testing in patients with metastatic colorectal cancer?

**Findings:**

In this retrospective cohort study of 41 061 patients, factors such as older age, treatment at community facilities, lower educational level in area of residence, and treatment at South Central regional facilities were associated with a reduced likelihood of microsatellite instability and *KRAS* testing.

**Meaning:**

Findings of this study highlight the sociodemographic-based disparities in biomarker testing, which can inform the development of strategies that promote equity in cancer care and improve outcomes for underserved populations.

## Teaser

This cohort study identifies the factors associated with likelihood of microsatellite instability and *KRAS* biomarker testing among patients with metastatic colorectal cancer.

## Introduction

As the third most prevalent and second leading cause of cancer-related deaths in the US, colorectal cancer (CRC) poses a formidable challenge to the global health care community.^1^ Initial diagnosis reveals metastatic disease in approximately 20% of patients with CRC,^1^ and the outcome is poor, with a 5-year survival rate of 15.6%.^2^

Nevertheless, precision oncology has emerged as a transformative approach to managing metastatic CRC (mCRC), a factor in substantially improved outcomes.^3,4^ Genetic biomarkers, such as microsatellite instability (MSI) and somatic alteration in the *BRAF* and *RAS* oncogenes have been the mainstay of precision medicine. In particular, *KRAS*-positive alteration is reported to be present in 35% to 45% of CRC cases.^5^ Numerous clinical trials have shown that anti–epidermal growth factor receptor monoclonal antibodies combined with chemotherapy improved survival outcomes for patients with wild-type *KRAS* CRC tumors.^6^ Additionally, the MSI pathway encompasses approximately 15% to 20% of sporadic CRC and the majority of hereditary CRC.^7^ Immune checkpoint inhibitors, such as pembrolizumab and nivolumab, have demonstrated remarkable responses in patients with MSI-high CRC, offering a promising first-line treatment for mCRC.^8,9^

Due to the prevalence and importance of the mentioned genetic alterations, guidelines recommend comprehensive biomarker testing for all patients with mCRC.^10^ Unfortunately, the health care field is plagued by a lack of access to several disciplines of the care process. Multiple studies have identified socioeconomic and demographic disparities associated with inequitable survival outcomes for patients with CRC.^11,12^ Numerous factors support these disparities, including genetic and behavioral characteristics,^13,14^ limited access to guideline-recommended screening,^15,16^ and discrepancies in treatment access and quality.^17,18,19^ Furthermore, reports have been emerging of disparate genetic testing access and counseling for patients with CRC.^20,21^ However, few studies have assessed the national discrepancies in access to genomic testing in CRC.

Therefore, the era of precision medicine could play a role in the exacerbation of preexisting disparities and widening of the gap in outcomes across specific populations. Consequently, in this study, we sought to evaluate the socioeconomic and demographic inequities in MSI and *KRAS* biomarker testing among patients with mCRC and to explore the association of testing with overall survival (OS).

## Methods

### Data Source

Data were obtained from the National Cancer Database (NCDB), a hospital-based cancer registry that includes demographic, clinical, and pathological data on more than 70% of patients with cancer diagnosed in the US.^22^ In accordance with the Common Rule, this cohort study was exempt from ethics review and informed consent requirement because it used deidentified patient data. We followed the Strengthening the Reporting of Observational Studies in Epidemiology (STROBE) reporting guideline.

### Study Design

Patients diagnosed with CRC between January 1, 2010, and December 31, 2017, were identified. Only patients with clinical stage IV were included, as biomarker testing was initially recommended for patients with metastatic disease. Given that the NCDB only reports MSI and *KRAS* biomarker tests in CRC, patients with available documentation on the completion (or noncompletion) of either test were included. Patients diagnosed before January 1, 2010, were excluded since *KRAS* testing was not prevalent before that period. In 2018, the NCDB changed the MSI and *KRAS* test variables and, since then, no longer reports patients who did not undergo these tests. Patients aged 80 years or older were excluded since the decision to perform biomarker testing may have been altered by the choice of not administering systemic therapy. Accordingly, patients were classified based on the completion (or noncompletion) of either MSI or *KRAS* test.

### Demographic, Socioeconomic, and Clinical Variables

The MSI and *KRAS* testing variables were recorded as site-specific factor 7 and site-specific factor 9, respectively. The NCDB reports that the tests were performed at any time between the date of diagnosis and first-course treatment. Nevertheless, the exact timing of testing was not reported. Sociodemographic variables were included, such as age, sex, race, ethnicity, insurance type, median household income, facility type, facility location (eTable 1 in Supplement 1), distance to facility, geographic location (US Census division), and educational level in area of residence. Race and ethnicity variables recorded in the NCDB were self-identified social constructs reported in the patients’ medical records. Educational level was assessed at the zip code level based on the percentage of adult residents in the area without a high school diploma. Median household income was also estimated by matching the zip code of the patient’s residence with files derived from the American Community Survey data. Clinical factors included Charlson Comorbidity Index, administration of chemotherapy and immunotherapy, surgery of the primary and nonprimary sites, and lung or liver metastases. A definition of socioeconomic and demographic factors is provided in eAppendix in Supplement 1.

### Statistical Analysis

The χ^2^ test assessed the socioeconomic and clinical categorical characteristic differences among the cohorts. Univariable and multivariable logistic regressions were conducted to determine the relevant independent factors associated with MSI and *KRAS* testing. Relative risk (RR) ratios and 95% CIs were calculated using Poisson models with robust covariance. A patient was considered to be positive for the event or outcome if the patient received biomarker testing. Univariable and multivariable Cox proportional hazards regression models were used to identify significant survival factors. The inclusion of clinical variables, such as guideline-recommended treatment options in the multivariable model, enabled the objective assessment of the association between testing and OS. Kaplan-Meier curves were produced based on test completion. The survival follow-up time was calculated from the date of diagnosis to the date of last contact or all-cause death. Due to missing date of testing, the date of diagnosis was used as the start of the follow-up period for the survival analysis. Treatment interventions were initiated within a short period after diagnosis and testing. The relatively short period between diagnosis, testing, and treatment, as defined by the NCDB, partially accounted for immortal time bias. Patients with missing follow-up data were not included in the survival analysis.

Two-sided *P* < .05 was considered to be statistically significant. The study was conducted between November 2022 and March 2024. SAS, version 9.4 (SAS Institute Inc), and R, version 4.2.3 (R Project for Statistical Computing), were used to analyze the data.

## Results

A total of 41 061 patients (22 362 males [54.5%], 18 699 females [45.5%]; mean [SD] age, 62.3 [10.1] years) diagnosed with mCRC between 2010 and 2017 were included (Figure). Of these patients, only 28.8% underwent *KRAS* testing and 43.7% underwent MSI testing. The population consisted of individuals who identified as Black (17.3%), White (78.0%), or other (4.7%) race with Hispanic (6.5%) or non-Hispanic (93.5%) ethnicity and who lived in a metropolitan setting (85.0%). Additionally, a substantial proportion of patients were covered by Medicare (43.6%), treated at a comprehensive community cancer program (40.5%) and South Atlantic facilities (21.3%), and resided in an area with lower educational level (51.3%). The clinical and sociodemographic characteristics of patients are shown in Table 1.

**Figure.**
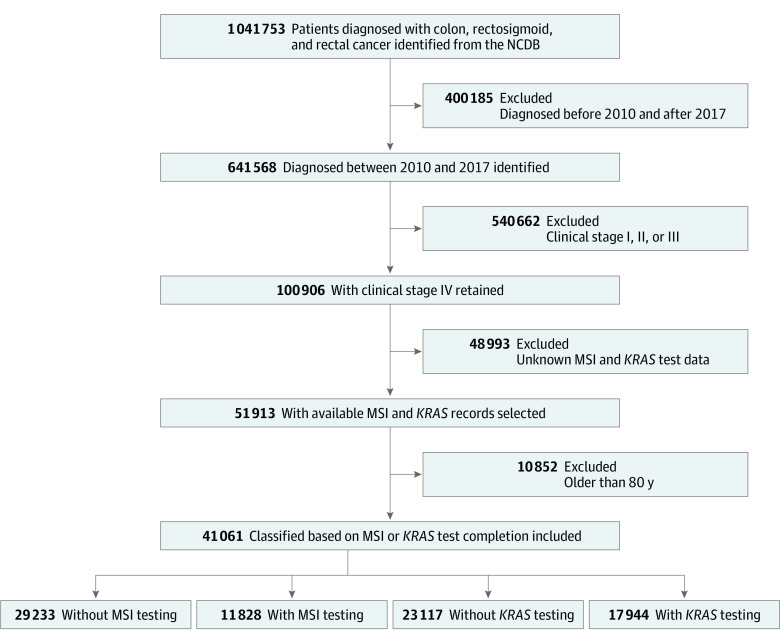
Study Flow Diagram MSI indicates microsatellite instability; NCDB, National Cancer Database.

**Table 1.** Baseline Sociodemographic and Clinical Characteristics Between Patients With and Without Biomarker Testing

Characteristic	MSI testing, No. (%)		*P* value	*KRAS* testing, No. (%)		*P* value
	Without (n = 29 233)	With (n = 11 828)		Without (n = 23 117)	With (n = 17 944)	
Age group, y						
18-49	2981 (57.0)	2249 (43.0)	<.001	2296 (43.9)	2934 (56.1)	<.001
50-59	7326 (67.5)	3529 (32.5)		5566 (51.3)	5289 (48.7)	
60-69	9800 (73.6)	3517 (26.4)		7582 (56.9)	5735 (43.1)	
70-79	9126 (78.3)	2533 (21.7)		7673 (65.8)	3986 (34.2)	
Race^a^						
Black	5297 (74.7)	1793 (25.3)	<.001	4156 (58.6)	2934 (41.4)	<.001
White	22 648 (70.7)	9406 (29.3)		17 902 (55.8)	14 152 (44.2)	
Other^a^	1288 (67.2)	629 (32.8)		1059 (55.2)	858 (44.8)	
Ethnicity^b^						
Hispanic^b^	1882 (70.6)	784 (29.4)	.48	1509 (56.6)	1157 (43.4)	.75
Non-Hispanic^a^	27 351 (71.2)	11 044 (28.8)		21 608 (56.3)	16 787 (43.7)	
Sex						
Male	16 057 (71.8)	6305 (28.2)	.003	12 533 (56.0)	9829 (44.0)	.26
Female	13 176 (70.5)	5523 (29.5)		10 584 (56.6)	8115 (43.4)	
Insurance type						
Private	10 346 (64.6)	5680 (35.4)	<.001	7810 (48.7)	8216 (51.3)	<.001
No insurance	1848 (76.6)	566 (23.4)		1450 (60.1)	964 (39.9)	
Medicaid	3001 (70.3)	1269 (29.7)		2416 (56.6)	1854 (43.4)	
Medicare or other^c^	13 675 (76.5)	4210 (23.5)		11 155 (62.4)	6730 (37.6)	
Unknown	363 (1.2)	103 (0.9)		286 (61.4)	180 (38.6)	
Educational level in area of residence, %^d^						
≥17.6	7457 (75.3)	2452 (24.7)	<.001	5961 (60.2)	3948 (39.8)	<.001
10.9-17.5	8243 (73.8)	2922 (26.2)		6564 (58.8)	4601 (41.2)	
6.3-10.8	7682 (69.3)	3396 (30.7)		6027 (54.4)	5051 (45.6)	
<6.3	5582 (65.5)	2944 (34.5)		4364 (51.2)	4162 (48.8)	
Unknown	269 (70.2)	114 (29.8)		201 (52.5)	182 (47.5)	
Facility type with CP						
Community	2885 (78.4)	796 (21.6)	<.001	2236 (60.7)	1445 (39.3)	<.001
Comprehensive	12 344 (74.1)	4304 (25.9)		9601 (57.7)	7047 (42.3)	
Academic	8710 (67.8)	4134 (32.2)		6895 (53.7)	5949 (46.3)	
Integrated network	5294 (67.1)	2594 (32.9)		4385 (55.6)	3503 (44.4)	
Median household income, US$						
<40 227	6689 (76.0)	2118 (24.0)	<.001	5364 (60.9)	3443 (39.1)	<.001
40 227-50 353	6784 (73.3)	2466 (26.7)		5367 (58.0)	3883 (42.0)	
50 354-63 332	6602 (70.6)	2753 (29.4)		5184 (55.4)	4171 (44.6)	
≥63 333	8864 (67.0)	4369 (33.0)		6982 (52.8)	6251 (47.2)	
Area of residence						
Metropolitan	24 681 (70.7)	10 237 (29.3)	<.001	19 535 (55.9)	15 383 (44.1)	.003
Urban	4051 (73.5)	1461 (26.5)		3217 (58.4)	2295 (41.6)	
Rural	501 (79.4)	130 (20.6)		365 (57.8)	266 (42.2)	
Facility location^e^						
New England	1235 (63.5)	710 (36.5)	<.001	1004 (51.6)	941 (48.4)	<.001
Middle Atlantic	4964 (71.1)	2018 (28.9)		3878 (55.5)	3104 (44.5)	
South Atlantic	6356 (72.6)	2403 (27.4)		5007 (57.2)	3752 (42.8)	
East North Central	5259 (72.5)	1992 (27.5)		4198 (57.9)	3053 (42.1)	
East South Central	1877 (75.1)	622 (24.9)		1567 (62.7)	932 (37.3)	
West North Central	2206 (71.6)	877 (28.4)		1591 (51.6)	1492 (48.4)	
West South Central	2760 (75.2)	912 (24.8)		2198 (59.9)	1474 (40.1)	
Mountain	1268 (66.4)	642 (33.6)		1012 (53.0)	898 (47.0)	
Pacific	3308 (66.7)	1652 (33.3)		2662 (53.7)	2298 (46.3)	
Travel distance, miles						
≤5	9877 (73.9)	3486 (29.5)	<.001	7821 (58.5)	5542 (41.5)	<.001
5.1-10	6655 (71.0)	2712 (29.0)		5220 (55.7)	4147 (44.3)	
10.1-25	7024 (69.9)	3019 (30.1)		5503 (54.8)	4540 (45.2)	
>25	5677 (68.5)	2611 (31.5)		4573 (55.2)	3715 (44.8)	
Year of diagnosis						
2010	4022 (85.8)	665 (14.2)	<.001	3145 (67.1)	1542 (32.9)	<.001
2011	3831 (81.6)	865 (18.4)		2886 (61.5)	1810 (38.5)	
2012	4214 (79.3)	1101 (20.7)		3085 (58.0)	2230 (42.0)	
2013	4285 (76.1)	1348 (23.9)		3109 (55.2)	2524 (44.8)	
2014	3891 (73.0)	1441 (27.0)		2887 (54.1)	2445 (45.9)	
2015	3466 (66.7)	1733 (33.3)		2776 (53.4)	2423 (46.6)	
2016	3029 (58.7)	2131 (41.3)		2633 (51.0)	2527 (49.0)	
2017	2495 (49.5)	2544 (50.5)		2596 (51.5)	2443 (48.5)	
CCI						
0	21 081 (70.4)	8867 (29.6)	<.001	16 459 (55.0)	13 489 (45.0)	<.001
1	5608 (72.8)	2100 (27.2)		4429 (57.5)	3279 (42.5)	
≥2	2544 (74.7)	861 (25.3)		2229 (65.5)	1176 (34.5)	
Grade						
Well-differentiated	1206 (66.8)	600 (33.2)	<.001	967 (53.5)	839 (46.5)	<.001
Moderately differentiated	11 607 (63.9)	6545 (36.1)		9159 (50.5)	8993 (49.5)	
Poorly differentiated	5577 (65.2)	2981 (34.8)		4572 (53.4)	3986 (46.6)	
Unknown	10 843 (86.4)	1702 (13.6)		8419 (67.1)	4126 (32.9)	
Immunotherapy						
Not administered	25 301 (75.3)	8279 (24.7)	<.001	20 489 (61.0)	13 091 (39.0)	<.001
Administered	3854 (52.3)	3512 (47.7)		2565 (34.8)	4801 (65.2)	
Unknown	78 (67.8)	37 (32.2)		63 (54.8)	52 (45.2)	
Chemotherapy						
Not administered	9449 (84.3)	1762 (15.7)	<.001	9104 (81.2)	2107 (18.8)	<.001
Administered	18 868 (65.9)	9772 (34.1)		13 195 (46.1)	15 445 (53.9)	
Unknown	916 (75.7)	294 (24.3)		818 (67.6)	392 (32.4)	
Liver metastasis at presentation						
Absent	6351 (71.6)	2524 (28.4)	<.001	5393 (60.8)	3482 (39.2)	<.001
Present	22 643 (71.0)	9258 (29.0)		17 529 (54.9)	14 372 (45.1)	
Unknown	570 (79.1)	151 (20.9)		467 (65.8)	254 (35.2)	
Lung metastasis at presentation						
Absent	21 130 (69.5)	9266 (30.5)	<.001	16 938 (55.7)	13 458 (44.3)	<.001
Present	7533 (75.8)	2411 (24.2)		5712 (57.4)	4232 (42.6)	
Unknown	570 (79.1)	151 (20.9)		467 (65.8)	254 (35.2)	
Surgery of primary site						
No	18 107 (85.0)	3190 (15.0)	<.001	13 564 (63.7)	7733 (36.3)	<.001
Yes	11 075 (56.2)	8625 (43.8)		9508 (48.3)	10 192 (51.7)	
Unknown	51 (79.7)	13 (20.3)		45 (70.3)	19 (29.7)	
Surgery of nonprimary site						
No	25 093 (74.8)	8474 (25.2)	<.001	19 658 (58.6)	13 909 (41.4)	<.001
Yes	4113 (55.2)	3342 (44.8)		3441 (46.2)	4014 (53.8)	
Unknown	27 (69.2)	12 (30.8)		18 (46.2)	21 (3.8)	

Abbreviations: CCI, Charlson Comorbidity Index (range: 0-25, with the highest score indicating a higher number of comorbidities); CP, cancer program; MSI, microsatellite instability.

^a^ Race data obtained from the National Cancer Database (NCDB) were self-identified in the patients’ medical records. Other races and non-Hispanic ethnicities include American Indian, Aleutian, or Eskimo; Asian Indian; Asian Indian or Pakistani; Chamorran; Chinese; Filipino; Fiji Islander; Guamanian; Hawaiian; Hmong; Japanese; Kampuchean; Korean; Laotian; Melanesian; Micronesian; New Guinean; Other; Other Asian; Pacific Islander; Polynesian; Samoan; Tahitian; Thai; Tongan; Unknown origin; and Vietnamese.

^b^ Ethnicity data obtained from the NCDB were self-identified in the patients’ medical records. Hispanic ethnicity includes Mexican, Puerto Rican, Cuban, South or Central American (except Brazil), Dominican Republic, or Spanish origin.

^c^ Other insurance type includes other governmental insurance.

^d^ Educational level was assessed at the zip code level based on the percentage of adult residents without a high school diploma.

^e^ US Census division.

Relative risk estimates for the event of MSI testing revealed factors associated with a lower likelihood of MSI testing (Table 2). Older patients had lower odds of MSI testing, with the lowest odds in the age group 70 to 79 years (RR, 0.70; 95% CI, 0.66-0.74; *P* < .001), followed by the 60-to-69-year (RR, 0.75; 95% CI, 0.72-0.78; *P* < .001) and 50-to-59-year (RR, 0.84; 95% CI, 0.81-0.87; *P* < .001) groups, compared with the age group of 18 to 49 years. Other significant factors included living in an area where the percentage of adults with no high school diploma was 17.6% or greater compared with an area with less than 6.3% (RR, 0.84; 95% CI, 0.79-0.89; *P* < .001), treatment at a community vs an academic cancer program (RR, 0.74; 95% CI, 0.70-0.79; *P* < .001), rural vs metropolitan residency (RR, 0.80; 95% CI, 0.69-0.92; *P* < .001), East South Central vs New England facilities (RR, 0.67; 95% CI, 0.61-0.73; *P* < .001). The association of factors with MSI testing in the univariable analysis is reported in eTable 2 in Supplement 1.

**Table 2.** Multivariable Logistic Regression Results With Relative Risk Estimates for Microsatellite Instability Testing

Characteristics	MSI testing		
	OR (95% CI)	*P* value	RR (95% CI)
Age group, y			
18-49	1 [Reference]	NA	NA
50-59	0.68 (0.63-0.73)	<.001	0.84 (0.81-0.87)
60-69	0.55 (0.50-0.59)	<.001	0.75 (0.72-0.78)
70-79	0.50 (0.45-0.55)	<.001	0.70 (0.66-0.74)
Race^a^			
White	1 [Reference]	NA	NA
Black	0.97 (0.90-1.04)	.35	0.98 (0.94-1.02)
Other^a^	0.98 (0.87-1.10)	.73	1.00 (0.94-1.06)
Ethnicity^b^			
Non-Hispanic^a^	1 [Reference]	NA	NA
Hispanic^b^	0.97 (0.87-1.07)	.51	0.98 (0.93-1.04)
Sex			
Male	1 [Reference]	NA	NA
Female	1.06 (1.01-1.12)	.02	1.04 (1.01-1.06)
Insurance type			
Private	1 [Reference]	NA	NA
No insurance	0.88 (0.78-0.98)	.03	0.93 (0.86-1.00)
Medicaid	0.89 (0.82-0.97)	.008	0.94 (0.90-0.99)
Medicare or other^c^	0.89 (0.83-0.95)	<.001	0.94 (0.90-0.97)
Unknown	0.67 (0.53-0.87)	.002	0.80 (0.68-0.93)
Educational level in area of residence, %^d^			
<6.3	1 [Reference]	NA	NA
6.3-10.8	0.89 (0.83-0.96)	.003	0.94 (0.91-0.98)
10.9-17.5	0.76 (0.69-0.82)	<.001	0.86 (0.82-0.90)
≥17.6	0.72 (0.65-0.79)	<.001	0.84 (0.79-0.89)
Unknown	1.35 (0.52-3.52)	.54	1.20 (0.66-2.18)
Facility type with CP			
Academic	1 [Reference]	NA	NA
Community	0.59 (0.53-0.65)	<.001	0.74 (0.70-0.79)
Comprehensive	0.67 (0.63-0.71)	<.001	0.81 (0.78-0.84)
Integrated network	1.02 (0.95-1.10)	.007	1.01 (0.98-1.05)
Area of residence			
Metropolitan	1 [Reference]	NA	NA
Urban	0.97 (0.88-1.05)	.42	0.98 (0.93-1.03)
Rural	0.67 (0.54-0.84)	<.001	0.80 (0.69-0.92)
Facility location^e^			
New England	1 [Reference]	NA	NA
Middle Atlantic	0.65 (0.58-0.74)	<.001	0.81 (0.76-0.86)
South Atlantic	0.55 (0.48-0.62)	<.001	0.73 (0.69-0.78)
East North Central	0.55 (0.49-0.62)	<.001	0.74 (0.69-0.79)
East South Central	0.46 (0.39-0.53)	<.001	0.67 (0.61-0.73)
West North Central	0.53 (0.46-0.61)	<.001	0.72 (0.67-0.77)
West South Central	0.50 (0.44-0.58)	<.001	0.70 (0.65-0.75)
Mountain	0.77 (0.66-0.90)	.001	0.88 (0.82-0.95)
Pacific	0.82 (0.72-0.93)	.002	0.90 (0.84-0.95)
Travel distance, miles			
≤5	1 [Reference]	NA	NA
5.1-10	1.07 (1.00-1.15)	.04	1.04 (1.00-1.08)
10.1-25	1.06 (0.99-1.14)	.09	1.02 (0.99-1.06)
>25	1.20 (1.10-1.30)	<.001	1.09 (1.05-1.14)
Year of diagnosis			
2010	1 [Reference]	NA	NA
2011	1.47 (1.31-1.65)	<.001	1.32 (1.21-1.44)
2012	1.80 (1.61-2.01)	<.001	1.51 (1.39-1.64)
2013	2.04 (1.82-2.28)	<.001	1.67 (1.54-1.81)
2014	2.59 (2.31-2.89)	<.001	1.94 (1.79-2.10)
2015	3.84 (3.44-4.30)	<.001	2.41 (2.24-2.61)
2016	5.78 (5.17-6.46)	<.001	2.94 (2.73-3.17)
2017	9.95 (8.89-11.14)	<.001	3.71 (3.45-4.00)
Median household income, US$			
≥63 333	1 [Reference]	NA	NA
50 354-63 332	1.02 (0.95-1.10)	.64	1.01 (0.97-1.05)
40 227-50 353	0.96 (0.88-1.04)	.28	0.97 (0.93-1.02)
<40 277	0.96 (0.87-1.06)	.44	0.97 (0.92-1.03)
Unknown	0.62 (0.25-1.58)	.32	0.77 (0.43-1.37)
CCI			
0	1 [Reference]	NA	NA
1	1.09 (1.02-1.16)	.01	1.04 (1.01-1.08)
≥2	1.11 (1.01-1.22)	.03	1.05 (1.00-1.11)
Immunotherapy			
Not administered	1 [Reference]	NA	NA
Administered	1.51 (1.42-1.62)	<.001	1.19 (1.15-1.23)
Unknown	1.41 (0.89-2.23)	.15	1.19 (0.92-1.53)
Chemotherapy			
Not administered	1 [Reference]	NA	NA
Administered	1.76 (1.65-1.88)	<.001	1.48 (1.42-1.55)
Unknown	1.28 (1.09-1.50)	.002	1.23 (1.11-1.36)
Surgery primary site			
No	1 [Reference]	NA	NA
Yes	5.34 (5.05-5.65)	<.001	2.80 (2.70-2.90)
Unknown	1.20 (0.63-2.30)	.59	1.18 (0.75-1.85)
Surgery nonprimary site			
No	1 [Reference]	NA	NA
Yes	1.23 (1.15-1.31)	<.001	1.08 (1.05-1.12)
Unknown	0.58 (0.28-1.22)	.15	0.78 (0.49-1.22)

Abbreviations: CCI, Charlson Comorbidity Index (range: 0-25, with the highest score indicating a higher number of comorbidities); CP, cancer program; MSI, microsatellite instability; NA, not applicable; OR, odds ratio; RR, relative risk.

^a^ Race data obtained from the National Cancer Database (NCDB) were self-identified in the patients’ medical records. Other races and non-Hispanic ethnicities include American Indian, Aleutian, or Eskimo; Asian Indian; Asian Indian or Pakistani; Chamorran; Chinese; Filipino; Fiji Islander; Guamanian; Hawaiian; Hmong; Japanese; Kampuchean; Korean; Laotian; Melanesian; Micronesian; New Guinean; Other; Other Asian; Pacific Islander; Polynesian; Samoan; Tahitian; Thai; Tongan; Unknown origin; and Vietnamese.

^b^ Ethnicity data obtained from the NCDB were self-identified in the patients’ medical records. Hispanic ethnicity includes Mexican, Puerto Rican, Cuban, South or Central American (except Brazil), Dominican Republic, or Spanish origin.

^c^ Other insurance type includes other governmental medical services, such as TRICARE, military, and Veterans Affairs.

^d^ Educational level was assessed at the zip code level based on the percentage of adult residents without a high school diploma.

^e^ US Census division.

For the event of *KRAS* testing, older patients (aged 70-79 years) had the lowest likelihood for testing compared with the group aged 18 to 49 years (RR, 0.81; 95% CI, 0.78-0.84; *P* < .001). Other factors associated with a lower likelihood for *KRAS* testing (Table 3) included living in an area where the percentage of adults with no high school diploma was 17.6% or greater compared with an area with less than 6.3% (RR, 0.92; 95% CI, 0.88-0.96; *P* < .001), Medicaid compared with private insurance (RR, 0.94; 95% CI, 0.91-0.98; *P* < .001), treatment at a community compared with an academic cancer program (RR, 0.92; 95% CI, 0.88-0.96; *P* < .001), and East South Central facilities compared with New England facilities (RR, 0.78; 95% CI, 0.73-0.83; *P* < .001). The association of factors with *KRAS* testing in the univariable analysis is reported in eTable 3 in Supplement 1.

**Table 3.** Multivariable Logistic Regression Results With Relative Risk Estimates for *KRAS* Testing

Characteristics	*KRAS* testing		
	OR (95% CI)	*P* value	RR (95% CI)
Age group, y			
18-49	1 [Reference]	NA	NA
50-59	0.84 (0.78-0.90)	<.001	0.93 (0.90-0.96)
60-69	0.77 (0.71-0.83)	<.001	0.90 (0.87-0.92)
70-79	0.64 (0.59-0.70)	<.001	0.81 (0.78-0.84)
Sex			
Male	1 [Reference]	NA	NA
Female	0.97 (0.93-1.02)	.21	0.99 (0.97-1.01)
Race^a^			
White	1 [Reference]	NA	NA
Black	1.05 (0.99-1.12)	.11	1.02 (0.99-1.06)
Other^a^	0.93 (0.84-1.03)	.14	0.97 (0.92-1.01)
Ethnicity^b^			
Non-Hispanic^a^	1 [Reference]	NA	NA
Hispanic^b^	1 (0.92-1.10)	<.001	1.00 (0.96-1.05)
Educational level in area of residence, %^c^			
<6.3	1 [Reference]	NA	NA
6.3-10.8	0.95 (0.89-1.01)	.10	0.98 (0.95-1.01)
10.9-17.5	0.85 (0.79-0.92)	<.001	0.93 (0.90-0.96)
≥17.6	0.84 (0.77-0.92)	<.001	0.92 (0.88-0.96)
Unknown	1.12 (0.51-2.48)	.78	1.06 (0.72-1.54)
Insurance type			
Private	1 [Reference]	NA	NA
No insurance	0.91 (0.83-1.00)	.06	0.96 (0.91-1.01)
Medicaid	0.88 (0.81-0.94)	<.001	0.94 (0.91-0.98)
Medicare or other^d^	0.93 (0.87-0.98)	.01	0.97 (0.94-0.99)
Unknown	0.83 (0.68-1.02)	.07	0.91 (0.82-1.02)
Facility type with CP			
Academic	1 [Reference]	NA	NA
Community	0.85 (0.78-0.92)	<.001	0.92 (0.88-0.96)
Comprehensive	0.89 (0.85-0.94)	<.001	0.95 (0.92-0.97)
Integrated network	0.97 (0.91-1.04)	.36	0.99 (0.96-1.02)
Area of residence			
Metropolitan	1 [Reference]	NA	NA
Urban	1.02 (0.95-1.10)	.62	1.01 (0.97-1.05)
Rural	1.02 (0.85-1.22)	.87	1.01 (0.92-1.11)
Facility location^e^			
New England	1 [Reference]	NA	NA
Middle Atlantic	0.87 (0.78-0.97)	.01	0.94 (0.89-0.99)
South Atlantic	0.79 (0.71-0.88)	<.001	0.90 (0.85-0.94)
East North Central	0.74 (0.66-0.82)	<.001	0.87 (0.83-0.91)
East South Central	0.60 (0.52-0.68)	<.001	0.78 (0.73-0.83)
West North Central	0.92 (0.81-1.04)	.19	0.96 (0.91-1.02)
West South Central	0.78 (0.69-0.88)	<.001	0.89 (0.84-0.94)
Mountain	0.99 (0.87-1.15)	.97	1.00 (0.94-1.07)
Pacific	0.98 (0.88-1.10)	.75	0.99 (0.94-1.05)
Travel distance, miles			
≤5	1 [Reference]	NA	NA
5.1-10	1.03 (0.97-1.09)	.28	1.01 (0.99-1.04)
10.1-25	0.99 (0.94-1.05)	.84	1.00 (0.97-1.02)
>25	0.99 (0.93-1.07)	.93	1.00 (0.96-1.03)
Year of diagnosis			
2010	1 [Reference]	NA	NA
2011	1.30 (1.19-1.42)	<.001	1.16 (1.10-1.22)
2012	1.52 (1.40-1.66)	<.001	1.26 (1.20-1.33)
2013	1.54 (1.41-1.68)	<.001	1.27 (1.21-1.34)
2014	1.62 (1.49-1.77)	<.001	1.31 (1.25-1.37)
2015	1.69 (1.54-1.84)	<.001	1.33 (1.27-1.40)
2016	1.78 (1.63-1.95)	<.001	1.36 (1.30-1.43)
2017	1.79 (1.64-1.96)	<.001	1.37 (1.30-1.44)
Median household income, US$			
≥63 333	1 [Reference]	NA	NA
50 354-63 332	1.03 (0.96-1.10)	.41	1.01 (0.98-1.04)
40 227-50 353	0.99 (0.92-1.06)	.74	0.99 (0.96-1.03)
<40 277	0.97 (0.89-1.05)	.41	0.98 (0.94-1.02)
Unknown	0.97 (0.45-2.07)	.93	0.98 (0.68-1.42)
CCI			
0	1 [Reference]	NA	NA
1	1.08 (1.02-1.14)	.008	1.04 (1.01-1.07)
≥2	0.92 (0.84-0.99)	.03	0.95 (0.91-0.99)
Immunotherapy			
Not administered	1 [Reference]	NA	NA
Administered	1.68 (1.59-1.79)	<.001	1.22 (1.19-1.25)
Unknown	1.32 (0.89-1.96)	.17	1.14 (0.95-1.37)
Chemotherapy			
Not administered	1 [Reference]	NA	NA
Administered	3.77 (3.56-3.99)	<.001	2.43 (2.34-2.54)
Unknown	1.82 (1.59-2.08)	<.001	1.60 (1.46-1.75)
Surgery primary site			
No	1 [Reference]	NA	NA
Yes	1.61 (1.54-1.68)	<.001	1.26 (1.24-1.29)
Unknown	0.72 (0.41-1.26)	.25	0.82 (0.57-1.18)
Surgery nonprimary site			
No	1 [Reference]	NA	NA
Yes	1.14 (1.07-1.20)	<.001	1.05 (1.02-1.08)
Unknown	1.18 (0.61-2.31)	.62	1.09 (0.84-1.41)

Abbreviations: CCI, Charlson Comorbidity Index (range: 0-25, with the highest score indicating a higher number of comorbidities); CP, cancer program; NA, not applicable; OR, odds ratio; RR, relative risk.

^a^ Race data obtained from the National Cancer Database (NCDB) were self-identified in the patients’ medical records. Other races and non-Hispanic ethnicities include American Indian, Aleutian, or Eskimo; Asian Indian; Asian Indian or Pakistani; Chamorran; Chinese; Filipino; Fiji Islander; Guamanian; Hawaiian; Hmong; Japanese; Kampuchean; Korean; Laotian; Melanesian; Micronesian; New Guinean; Other; Other Asian; Pacific Islander; Polynesian; Samoan; Tahitian; Thai; Tongan; Unknown origin; and Vietnamese.

^b^ Ethnicity data obtained from the NCDB were self-identified in the patients’ medical records. Hispanic ethnicity includes Mexican, Puerto Rican, Cuban, South or Central American (except Brazil), Dominican Republic, or Spanish origin.

^c^ Educational level was assessed at the zip code level based on the percentage of adult residents without a high school diploma.

^d^ Other insurance type includes other governmental insurance.

^e^ US Census division.

Unadjusted survival analysis (eFigures 1 and 2 in Supplement 1) revealed that improved median OS for patients was associatedwith MSI testing vs without testing (23.89 [95% CI, 23.30-24.48] months vs 12.29 [95% CI, 12.01-12.57] months) and with *KRAS* testing vs without testing (20.40 [95% CI, 20.01-20.79] months vs 10.68 [95% CI, 10.34-11.02] months). The median (IQR) follow-up period for the survival analysis was 13.96 (3.71-29.34) months. In the multivariable Cox proportional hazards regression model, MSI testing (hazard ratio [HR], 0.93; 95% CI, 0.91-0.96; *P* < .001) and *KRAS* testing (HR, 0.97; 95% CI, 0.95-1.00; *P* = .03) were associated with a modest improvement in OS after adjusting for confounders compared with no MSI and *KRAS* testing. Patterns of increased MSI (14.2% to 50.5%) and *KRAS* (32.9% to 48.5%) testing are reported between 2010 and 2017, with a 9.2% increase in MSI testing compared with the 0.5% decrease in *KRAS* testing during the last year of the study (eFigure 3 in Supplement 1). Table 4 highlights the factors associated with OS in this patient cohort.

**Table 4.** Univariable and Multivariable Cox Proportional Hazards Regression Models for Overall Survival

Characteristics	Univariable analysis		Multivariable analysis	
	HR (95% CI)	*P* value	HR (95% CI)	*P* value
MSI testing				
No	1 [Reference]	NA	1 [Reference]	NA
Yes	0.62 (0.60-0.63)	<.001	0.93 (0.91-0.96)	<.001
*KRAS* testing				
No	1 [Reference]	NA	1 [Reference]	NA
Yes	0.71 (0.69-0.72)	<.001	0.97 (0.95-1.00)	.03
Age, y				
18-49	1 [Reference]	NA	1 [Reference]	NA
50-59	1.16 (1.12-1.20)	<.001	1.06 (1.02-1.10)	.007
60-69	1.43 (1.38-1.49)	<.001	1.16 (1.12-1.21)	<.001
70-79	1.90 (1.83-1.97)	<.001	1.30 (1.24-1.35)	<.001
Sex				
Male	1 [Reference]	NA	1 [Reference]	NA
Female	0.96 (0.94-0.98)	<.001	0.98 (0.96-1.00)	.07
Ethnicity^a^				
Non-Hispanic	1 [Reference]	NA	1 [Reference]	NA
Hispanic	0.83 (0.79-0.87)	<.001	0.84 (0.79-0.88)	<.001
CCI				
0	1 [Reference]	NA	1 [Reference]	NA
1	1.18 (1.15-1.21)	<.001	1.08 (1.05-1.11)	<.001
≥2	1.60 (1.54-1.66)	<.001	1.28 (1.24-1.34)	<.001
Grade				
Well-differentiated	1 [Reference]	NA	1 [Reference]	NA
Moderately differentiated	1.03 (0.97-1.08)	.40	1.12 (1.06-1.18)	<.001
Poorly differentiated	1.59 (1.50-1.69)	<.001	1.80 (1.70-1.91)	<.001
Insurance type				
Private	1 [Reference]	NA	1 [Reference]	NA
No insurance	1.38 (1.31-1.45)	<.001	1.20 (1.14-1.26)	<.001
Medicaid	1.26 (1.21-1.31)	<.001	1.14 (1.09-1.18)	<.001
Medicare or other^b^	1.56 (1.52-1.60)	<.001	1.13 (1.10-1.16)	<.001
Unknown	1.32 (1.19-1.47)	<.001	1.08 (0.98-1.20)	.14
Educational level in area of residence, %^c^				
≥17.6	1 [Reference]	NA	1 [Reference]	NA
10.9-17.5	1.03 (1.00-1.06)	.11	0.99 (0.96-1.03)	.62
6.3-10.8	0.97 (0.94-1.00)	.02	0.98 (0.94-1.02)	.29
Unknown	1.02 (0.91-1.15)	.69	1.06 (0.69-1.62)	.78
Facility type with CP				
Academic	1 [Reference]	NA	1 [Reference]	NA
Community	1.24 (1.19-1.29)	<.001	1.07 (1.02-1.12)	.003
Comprehensive	1.20 (1.17-1.23)	<.001	1.13 (1.10-1.17)	<.001
Integrated network	1.16 (1.13-1.20)	<.001	1.13 (1.09-1.16)	<.001
Median household income, US$				
≥63 333	1 [Reference]	NA	1 [Reference]	NA
50 354-63 332	1.09 (1.06-1.12)	<.001	1.05 (1.01-1.08)	.007
40 227-50 353	1.16 (1.12-1.19)	<.001	1.08 (1.04-1.12)	<.001
<40 277	1.20 (1.17-1.24)	<.001	1.10 (1.05-1.15)	<.001
Unknown	1.13 (1.01-1.26)	.04	0.97 (0.65-1.46)	.89
Year of diagnosis				
2010	1 [Reference]	NA	1 [Reference]	NA
2011	0.96 (0.92-1.00)	.07	0.97 (0.93-1.01)	.18
2012	0.93 (0.89-0.97)	<.001	0.92 (0.88-0.96)	<.001
2013	0.95 (0.91-0.99)	.01	0.96 (0.92-1.00)	.07
2014	0.96 (0.92-1.00)	.05	0.98 (0.94-1.02)	.31
2015	0.91 (0.87-0.95)	<.001	0.93 (0.89-0.97)	.002
2016	0.86 (0.82-0.90)	<.001	0.91 (0.87-0.95)	<.001
2017	0.81 (0.77-0.85)	<.001	0.83 (0.79-0.87)	<.001
Immunotherapy				
Not administered	1 [Reference]	NA	1 [Reference]	NA
Administered	0.65 (0.63-0.67)	<.001	0.85 (0.82-0.87)	<.001
Unknown	0.85 (0.68-1.05)	.13	0.97 (0.77-1.21)	.76
Chemotherapy				
Not administered	1 [Reference]	NA	1 [Reference]	NA
Administered	0.31 (0.30-0.32)	<.001	0.37 (0.36-0.38)	<.001
Unknown	0.41 (0.38-0.44)	<.001	0.49 (0.45-0.52)	<.001
Lung metastasis at presentation				
Absent	1 [Reference]	NA	1 [ Reference]	NA
Present	1.33 (1.30-1.37)	<.001	1.16 (1.13-1.19)	<.001
Unknown	1.51 (1.39-1.64)	<.001	1.07 (0.98-1.17)	.14
Liver metastasis at presentation				
Absent	1 [Reference]	NA	1 [Reference]	NA
Present	1.10 (1.07-1.13)	<.001	1.27 (1.23-1.30)	<.001
Unknown	1.52 (1.33-1.73)	<.001	1.09 (0.95-1.26)	.21
Surgery primary site				
No	1 [Reference]	NA	1 [Reference]	NA
Yes	0.46 (0.45-0.47)	<.001	0.53 (0.52-0.55)	<.001
Unknown	0.54 (0.39-0.74)	<.001	0.58 (0.42-0.82)	.002
Surgery nonprimary site				
No	1 [Reference]	NA	1 [Reference]	NA
Yes	0.56 (0.54-0.58)	<.001	0.81 (0.79-0.84)	<.001
Unknown	0.56 (0.38-0.83)	.003	0.78 (0.53-1.15)	.21
Area of residence				
Metropolitan	1 [Reference]	NA	1 [Reference]	NA
Urban	1.11 (1.08-1.15)	<.001	1.04 (1.00-1.08)	.07
Rural	1.16 (1.06-1.26)	.001	1.11 (1.02-1.22)	.02
Facility location^d^				
New England	1 [Reference]	NA	1 [Reference]	NA
Middle Atlantic	0.91 (0.86-0.96)	.001	0.85 (0.80-0.90)	<.001
South Atlantic	1.03 (0.98-1.09)	.28	1.02 (0.96-1.08)	.56
East North Central	1.08 (1.02-1.14)	.01	1.08 (1.02-1.14)	.008
East South Central	1.07 (1.00-1.15)	.04	1.03 (0.97-1.11)	.35
West North Central	1.03 (0.97-1.10)	.35	1.06 (1.00-1.13)	.07
West South Central	0.96 (0.90-1.02)	.21	0.84 (0.79-0.89)	<.001
Mountain	1.01 (0.94-1.09)	.73	0.94 (0.87-1.00)	.07
Pacific	0.98 (0.93-1.04)	.50	0.98 (0.92-1.04)	.45
Travel distance, miles				
≤5	1 [Reference]	NA	1 [Reference]	NA
5.1-10	0.94 (0.92-0.97)	<.001	0.98 (0.95-1.01)	.22
10.1-25	0.90 (0.87-0.93)	<.001	0.99 (0.97-1.03)	.75
>25	0.90 (0.87-0.93)	<.001	0.99 (0.96-1.03)	.71

Abbreviations: CCI, Charlson Comorbidity Index (range: 0-25, with the highest score indicating a higher number of comorbidities); CP, cancer program; HR, hazard ratio; MSI, microsatellite instability; NA, not applicable.

^a^ Ethnicity data obtained from the NCDB were self-identified in the patients’ medical records. Hispanic ethnicity includes Mexican, Puerto Rican, Cuban, South or Central American (except Brazil), Dominican Republic, or Spanish origin.

^b^ Other insurance type includes other governmental insurance.

^c^ Educational level was assessed at the zip code level based on the percentage of adult residents without a high school diploma.

^d^ US Census division.

## Discussion

This nationwide analysis found associations of socioeconomic and demographic factors with decreased MSI and *KRAS* testing. Sociodemographic factors, including community-setting treatment, treatment at East South Central facilities, lower educational levels in area of residence, and older age, were associated with a reduced likelihood of MSI and *KRAS* testing. Rural residence was associated with lower odds of MSI testing only. Slightly improved OS outcomes were reported for patients who underwent MSI tests.

Patients treated at community cancer programs and comprehensive community cancer programs had lower odds of receiving biomarker testing than those at academic programs. Academic facilities may be more likely to order biomarker testing due to their research focus, access to specialized resources, and involvement in clinical trials. A recent survey revealed that oncologists at academic centers were more likely to order biomarker testing at the time of initial biopsy than oncologists in community cancer programs (76% vs 52%) and to involve the patient’s family in biomarker testing discussions (85% vs 59%; *P* = .009).^23^ Furthermore, in a survey by the Association of Community Cancer Centers, over half of the respondents indicated their programs had no standard protocol for biomarker testing, whereas 14% were unsure if any protocols existed in their institutions.^24^ The same survey revealed that patients at these facilities required psychosocial support, genetic counseling, and financial assistance.^24^ Given that most US patients with cancer are treated in community settings, addressing methods to increase compliance with biomarker testing in community cancer programs is crucial.^25^

Moreover, patients residing in areas with lower educational level demonstrated lower MSI and *KRAS* testing rates, possibly due to a limited understanding of biomarker testing’s utility. While we could not assess linguistic abilities due to insufficient information in the NCDB and, although Hispanic ethnicity was not associated with a lack of testing, we contend that patients’ understanding of medical terminology and the purpose of testing were below physicians’ expectations. This disparity is exemplified in an analysis reporting that almost half of the patients could not describe specific terms related to biomarker testing immediately after an explanation by their oncologists.^26^ False perceptions toward biomarker testing can be attributed to lower health literacy levels and limited efforts by clinicians to explain comprehensively the importance of testing, both of which can be deterrents for patients with lower educational levels.

Patients residing in rural areas were less likely to receive biomarker testing. These patients could face logistic challenges in accessing specialized tertiary centers or diagnostic facilities that offer genetic testing. This finding aligns with the geographic association between US Census divisions and the outcome, where East South Central facilities had the lowest likelihood of MSI and *KRAS* testing, followed by West South Central compared with New England facilities. The widespread rural landscape in these regions may explain the association. According to data from the US Census Bureau, states such as Mississippi, Arkansas, Alabama, and Kentucky are among the top states with rural populations.^27^ It is also important to analyze rurality in the context of the vicinity of urban or metropolitan areas given that these states also have the lowest urban populations and are not in proximity to major urban cities. Additionally, national data recorded the lowest levels of bachelor’s degree attainment in South Central states, such as Kentucky, Louisiana, Arkansas, and Mississippi.^28^ These demographic factors may explain the lower rates of MSI and *KRAS* testing in these regions given the association of rurality and educational level with the primary outcome. We believe that this finding poses a serious dilemma because the Centers for Disease Control and Prevention reported that Mississippi and Kentucky in East South Central, along with Louisiana and Arkansas in West South Central, exhibited notably higher incident CRC cases than other states.^29^ Consequently, the same states had among the highest rates of CRC deaths over the years studied and up to the present date.^29^ Although granular state-specific data would assist in identifying the risk factors for the suboptimal outcomes, the lower likelihood for patients to undergo testing, compounded by the high incidence in these regions, may be associated with these worse outcomes.

Patients with late-onset disease (≥50 years) were less likely to undergo biomarker testing. This finding can be explained by the initial recommendation of MSI testing for younger patients with a hereditary component, such as Lynch syndrome. Prior to the US Food and Drug Administration approval of pembrolizumab for MSI-high tumors, MSI testing was predominantly performed to identify patients likely to have Lynch syndrome and to assess outcome.^30,31^ Alternatively, the gradual decrease in likelihood with the incremental increase in age groups for both tests may implicate the possible lack of compliance by physicians and/or patients. Patients with late-onset disease may not have been fully aware of the importance of MSI and *KRAS* testing due to the relative novelty of the field during the present study. The findings align with historical evidence of the increased acceptance of genetic testing in younger patients.^32^ It is plausible that concerns regarding the burdensome treatment after testing and its implications for quality of life may have deterred older patients, considering their high comorbidity score and limited life expectancy. On the other hand, physicians’ perceptions may have dissuaded them from testing older patients due to concerns about toxic effects and/or perceived limited effectiveness.

The results of the present study did not show an association with insurance, which may be interpreted in different ways. First, health coverage alone may not explain disparities in testing, and other factors can be considered. Second, access to private insurance and government insurance (Medicaid and Medicare) differs by state. Third, while some states had legislation mandating coverage for biomarker testing during the study period, others did not implement such legislation until 2021, suggesting that insurance did not play a crucial role in accessing biomarker testing during the study.^33^

While this study did not find any association between race and ethnicity and biomarker testing, evidence of racial and ethnic disparities is documented in the literature.^34^ The lack of significance after adjusting for covariates suggests that racial and ethnic disparities may be mediated, at least partially, through socioeconomic, demographic, and clinical variables. Moreover, race and ethnicity can serve as a precursor for a cascade of socioeconomic disparities. The multifaceted association among these variables confirms the complexity of drawing conclusions from analyses of social determinants of health.

### Clinical Implications

Survival benefits were associated with MSI and *KRAS* testing, as demonstrated by the Kaplan-Meier analysis and univariable Cox proportional hazards regression model. Nevertheless, the benefit was diluted when therapeutic interventions (and other factors) were accounted for in the multivariable analysis. The difference between the univariable and multivariable associations was the nature of the intervention itself given that testing guides therapeutic choices. These findings highlight the prominence of confounding clinical variables on survival outcomes and the importance of timely treatment follow-up after testing, given the abundant literature supporting the advantages of tailored therapeutics.^8,35,36,37,38^ Additionally, the significant difference between the unadjusted and adjusted models suggests that testing acts as a surrogate of overall cancer care, reflecting broader systemic disparities in treatment access and quality. Patients without biomarker testing can have a perpetual vulnerability of lack of access to other treatment options. In short, disparities in MSI and *KRAS* testing can compromise optimal mCRC treatment.

### Temporal Patterns of Biomarker Testing

Despite the apparent underuse of biomarkers, our study demonstrated a progressive increase in testing. The percentage of patients who were tested significantly increased from 32.9% to 48.5% for *KRAS* and from 14.2% to 50.5% for MSI. Although *KRAS* testing was integrated into national guidelines earlier than MSI testing, MSI testing became more prevalent than *KRAS* testing during the last year of the study, with a 9.2% increase in MSI testing and 0.5% decrease in *KRAS* testing between 2016 and 2017. The treatment landscape for mCRC has been rapidly expanding to include various MSI-tailored immunotherapies associated with substantially improved outcomes, more so than *KRAS*-guided treatments. Nevertheless, while these findings may be affected by historical and contemporary societal structural policies, the recent improvement in testing implies that familiarization with this novel field could help incorporate testing that is concordant with national recommendations.

### Limitations

The study’s retrospective nature did not account for nonobservable confounding variables. Moreover, data were extrapolated from the NCDB, which collects predesignated variables from hospitals, limiting the ability to report the specific timing of testing and the type of test used. Due to the unavailability of the exact time of testing, we considered the date of diagnosis as the start of the follow-up period for the survival analysis. Meanwhile, it is important to highlight the geographic context of educational level and median household incomes in the analysis. Additionally, the NCDB does not report patients who did not undergo the test after 2017, restricting the inclusion of patients with recent years of diagnosis. We also acknowledge that biomarker testing is a surrogate of overall care and that the heterogeneity of institutions within the NCDB is a factor in variations in quality of care. Nevertheless, using the NCDB, which encompasses the largest number of facilities and spans US geography, to investigate topics of disparities provides valuable insight into the implementation of clinical practice.

## Conclusions

In this cohort study of patients with mCRC, older age, lower educational level in area of residence, community-setting treatment, and treatment at East South Central facilities were associated with a lower likelihood of MSI and *KRAS* testing. By highlighting the sociodemographic-based disparities in biomarker testing using national registries, we can develop strategies for promoting equity in cancer care and improving outcomes for underserved populations. Further research is encouraged to assess biomarker testing at the state level rather than the regional level, with particular emphasis on sociodemographically vulnerable populations.

## Notes

Supplement 1. **eAppendix.** Definition of Socioeconomic and Demographic Factors **eFigure 1.** Kaplan Meier Overall Survival Curves Based on MSI Testing **eFigure 2.** Kaplan Meier Overall Survival Curves Based on KRAS Testing **eFigure 3.** Pattern of Biomarker Testing per Year of Diagnosis **eTable 1.** US Census Division of the Regions by States **eTable 2.** Univariable Association of Factors With MSI Testing **eTable 3.** Univariable Association of Factors With KRAS Testing
eReferences

Supplement 2.
Data Sharing Statement
