# Supplementary material for: Biomarker Testing Disparities in Metastatic Colorectal Cancer
Source: JAMA Netw Open. 2024 Jul 5;7(7):e2419142. doi: 10.1001/jamanetworkopen.2024.19142 (PMC11227072; doi:10.1001/jamanetworkopen.2024.19142)
Supplement: Supplement 1. — eAppendix. Definition of Socioeconomic and Demographic Factors eFigure 1. Kaplan Meier Overall Survival Curves Based on MSI Testing eFigure 2. Kaplan Meier Overall Survival Curves Based on KRAS Testing eFigure 3. Pattern of Biomarker Testing per Year of Diagnosis eTable 1. US Census Division of the Regions by States eTable 2. Univariable Association of Factors With MSI Testing eTable 3. Univariable Association of Factors With KRAS Testing eReferences [file jamanetwopen-e2419142-s001.pdf]

Supplemental Online Content

Sabbagh S, Herrán M, Hijazi A, et al. Biomarker testing disparities in metastatic colorectal cancer. *JAMA Netw Open*. 2024;7(7):e2419142. doi:10.1001/jamanetworkopen.2024.19142

eAppendix. Definition of Socioeconomic and Demographic Factors

eFigure 1. Kaplan Meier Overall Survival Curves Based on MSI Testing

eFigure 2. Kaplan Meier Overall Survival Curves Based on KRAS Testing

eFigure 3. Pattern of Biomarker Testing per Year of Diagnosis

eTable 1. US Census Division of the Regions by States

eTable 2. Univariable Association of Factors With MSI Testing

eTable 3. Univariable Association of Factors With KRAS Testing

eReferences

This supplemental material has been provided by the authors to give readers additional information about their work.

© 2024 Sabbagh S et al. *JAMA Network Open*

eAppendix. Definition of Socioeconomic and Demographic Factors

Socioeconomic variables have been defined as factors of a social and economic nature, such as income, education, employment status, and insurance coverage, which collectively serve as indicators of an individual's standing within a community[40]. Demographic variables indicate the characteristics of a population, including age, ethnicity, race, and sex. The area of residence can potentially be classified as demographic, socioeconomic, or even geographic. Meanwhile, facility location and travel distance are considered geographic factors.

© 2024 Sabbagh S et al. *JAMA Network Open*

eFigure 1. Kaplan Meier Overall Survival Curves Based on MSI Testing

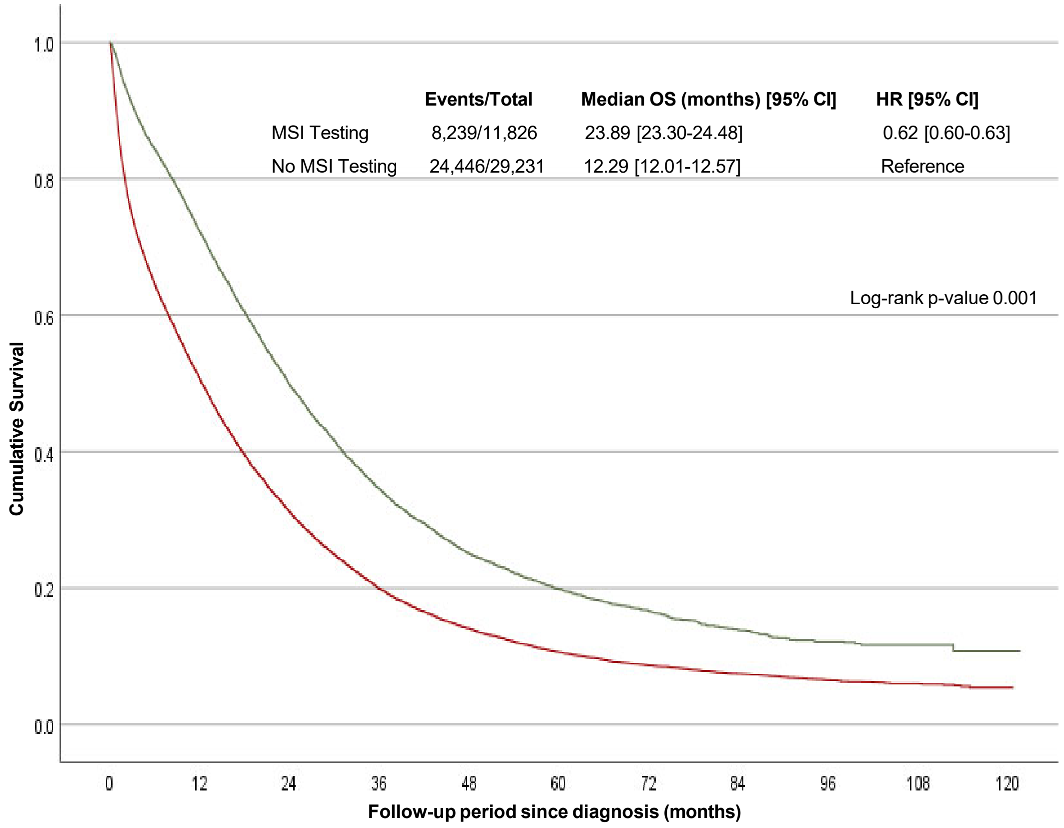

No. at risk											
**MSI Testing**	11,826	11,462	11,097	10,732	10,367	10,002	9,637	9,272	8,907	8,542	8,177
**No MSI Testing**	29,231	28,867	28,502	28,137	27,772	27,407	27,042	26,677	26,312	25,947	25,582

© 2024 Sabbagh S et al. *JAMA Network Open*

eFigure 2. Kaplan Meier Overall Survival Curves Based on KRAS Testing

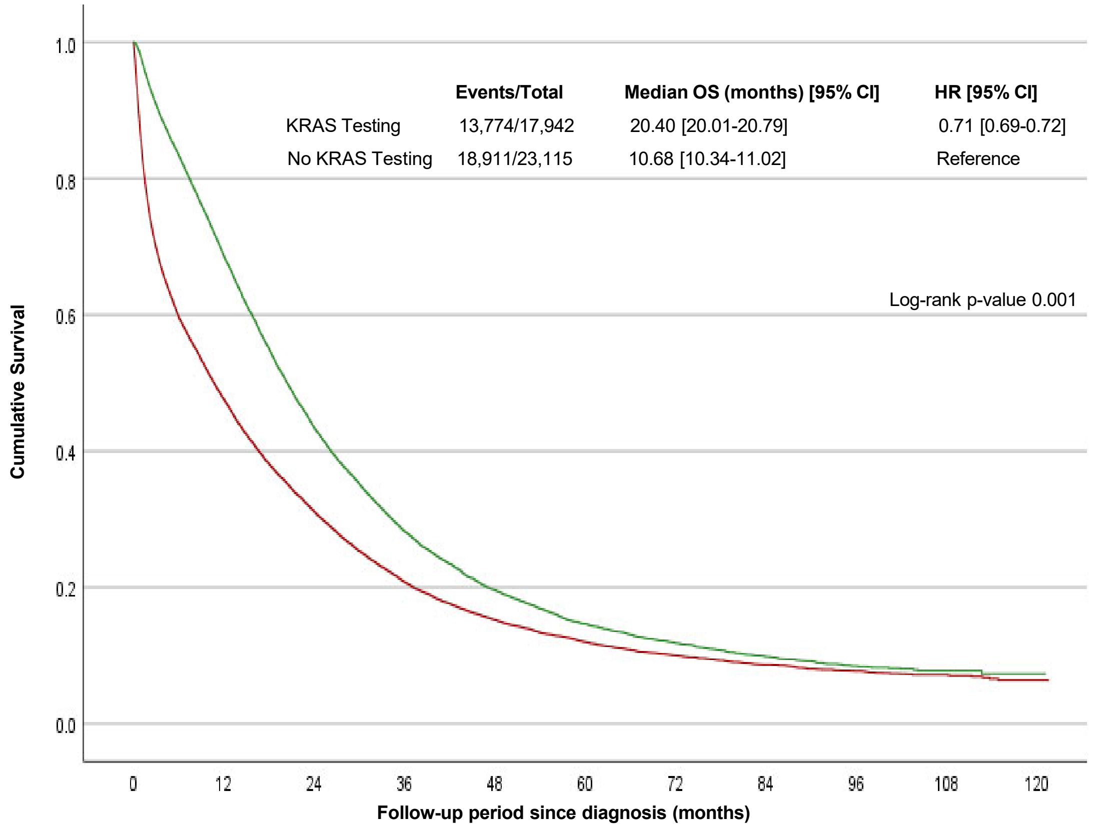

**No. at risk**											
**KRAS Testing**	17,942	17,578	17,213	16,848	16,483	16,118	15,753	15,388	15,023	14,658	14,293
**No KRAS Testing**	23,115	22,751	22,386	22,021	21,656	21,191	20,926	20,561	20,196	19,831	19,466

© 2024 Sabbagh S et al. *JAMA Network Open*

eFigure 3. Pattern of Biomarker Testing Per Year of Diagnosis

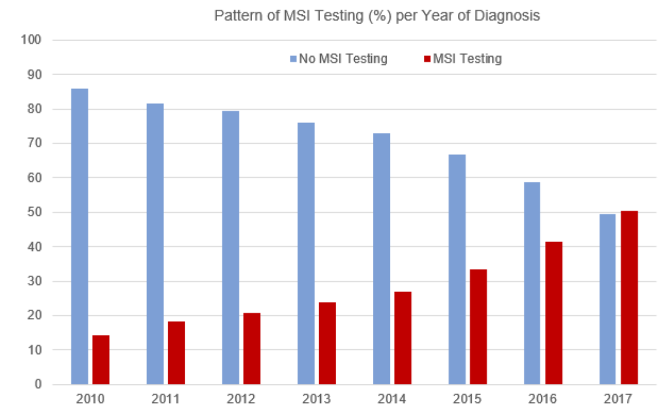

	2010	2011	2012	2013	2014	2015	2016	2017
**No MSI Testing (%)**	85.8	81.6	79.3	76.1	73.0	66.7	58.7	49.5
**MSI Testing (%)**	14.2	18.4	20.7	23.9	27.0	33.3	41.3	50.5

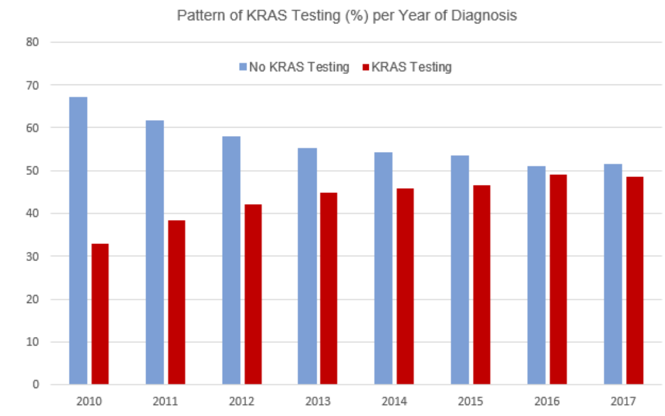

	2010	2011	2012	2013	2014	2015	2016	2017
**No KRAS Testing (%)**	67.1	61.5	58.0	55.2	54.1	53.4	51.0	51.5
**KRAS Testing (%)**	32.9	38.5	42.0	44.8	45.9	46.6	49.0	48.5

© 2024 Sabbagh S et al. *JAMA Network Open*

eTable 1. US Census Division of the Regions by States

REGION	STATES INCLUDED
**New England**	CT, MA, ME, NH, RI, VT
**Middle Atlantic**	NJ, NY, PA
**South Atlantic**	DC, DE, FL, GA, MD, NC, SC, VA, WV
**East North Central**	IL, IN, MI, OH, WI
**East South Central**	AL, KY, MS, TN
**West North Central**	IA, KS, MN, MO, ND, NE, SD
**West South Central**	AR, LA, OK, TX
**Mountain**	AZ, CO, ID, MT, NM, NV, UT, WY
**Pacific**	AK, CA, HI, OR, WA

© 2024 Sabbagh S et al. *JAMA Network Open*

eTable 2. Univariable Association of Factors with MSI Testing

Characteristics	Microsatellite Testing		p-value
	OR	95% CI	
**Age, years**			
18 - 49	1 [Reference]	–	–
50 - 59	0.64	(0.60 – 0.68)	<0.001
60 - 69	0.48	(0.45 – 0.51)	<0.001
70 - 79	0.37	(0.34 – 0.40)	<0.001
**Race**			
White	1 [Reference]	–	–
Black	0.82	(0.77 – 0.86)	<0.001
Others[a]	1.18	(1.07 – 1.30)	0.001
**Ethnicity**			
Non-Hispanic	1 [Reference]	–	–
Hispanic[b]	1.03	(0.95 – 1.13)	0.48
**Sex**			
Male	1 [Reference]	–	–
Females	1.07	(1.02 – 1.11)	0.003
**Insurance Status**			
Private insurance	1 [Reference]	–	–
Uninsured	0.56	(0.51 – 0.62)	<0.001
Medicaid	0.77	(0.72 – 0.83)	<0.001
Medicare/Other Governmental	0.56	(0.54 – 0.59)	<0.001
Unknown	0.52	(0.41 – 0.65)	<0.001
**Education**			
<6.3%	1 [Reference]	–	–
6.3%-10.8%	0.84	(0.79 – 0.89)	<0.001
10.9%-17.5%	0.67	(0.63 – 0.72)	<0.001
≥17.6%	0.62	(0.59 – 0.67)	<0.001
Unknown	0.80	(0.64 – 1.00)	0.06
**Facility type**			
Academic CP[a]	1 [Reference]	–	–
Community CP[a]	0.58	(0.53 – 0.63)	<0.001
Comprehensive CP[a]	0.74	(0.70 – 0.77)	<0.001
Integrated network CP[a]	1.03	(0.97 – 1.10)	0.30
**Area of Residence**			
Metro	1 [Reference]	-	-
Urban	0.87	(0.82 – 0.93)	<0.001
Rural	0.63	(0.52 – 0.76)	<0.001
**Facility Location**			
New England	1 [Reference]	–	–
Middle Atlantic	0.71	(0.64 – 0.79)	<0.001
South Atlantic	0.66	(0.59 – 0.73)	<0.001
East North Central	0.66	(0.59 – 0.73)	<0.001
East South Central	0.58	(0.51 – 0.66)	<0.001

© 2024 Sabbagh S et al. *JAMA Network Open*

Characteristics	Microsatellite Testing OR	95% CI	p-value
West North Central	0.69	(0.61 – 0.78)	<0.001
West South Central	0.58	(0.51 – 0.65)	<0.001
Mountain	0.88	(0.77 – 1.00)	0.06
Pacific	0.87	(0.78 – 0.97)	0.01
**Travel distance**			
≤5 miles	1 [Reference]	–	–
5.1-10	1.16	(1.09 – 1.23)	<0.001
10.1-25	1.22	(1.15 – 1.29)	<0.001
>25 miles	1.30	(1.23 – 1.38)	<0.001
**Year of Diagnosis**			
2010	1 [Reference]	–	–
2011	1.37	(1.22 – 1.53)	<0.001
2012	1.58	(1.42 – 1.76)	<0.001
2013	1.90	(1.72 – 2.11)	<0.001
2014	2.24	(2.02 – 2.48)	<0.001
2015	3.02	(2.74 – 3.34)	<0.001
2016	4.26	(3.85 – 4.70)	<0.001
2017	6.17	(5.59 – 6.81)	<0.001
**Median Household Income**			
≥$63,333	1 [Reference]	–	–
$50,354-$63,332	0.85	(0.8 – 0.9)	<0.001
$40,227-$50,353	0.74	(0.7 – 0.78)	<0.001
<$40,277	0.64	(0.6 – 0.68)	<0.001
Unknown	0.84	(0.68-1.04)	0.12
**Charlson Comorbidity Index**			
0	1 [Reference]	–	–
1	0.89	(0.84 – 0.94)	<0.001
≥2	0.81	(0.74 – 0.87)	<0.001
**Immunotherapy**			
Not Administered	1 [Reference]	–	–
Administered	2.79	(2.64 – 2.93)	<0.001
Unknown	1.45	(0.98 – 2.15)	0.06
**Chemotherapy**			
Not administered	1 [Reference]	–	–
Administered	2.78	(2.63 – 2.94)	<0.001
Unknown	1.72	(1.50 – 1.98)	<0.001
**Surgery Primary Site**			
No	1 [Reference]	–	–
Yes	4.42	(4.22 – 4.63)	<0.001
Unknown	1.45	(0.79 – 2.66)	0.24
**Surgery Non-Primary Site**			
No	1 [Reference]	–	–
Yes	2.41	(2.28 – 2.53)	<0.001
Unknown	1.32	(0.67 – 2.60)	0.43

CCI: Charlson Comorbidity Index, CP: Cancer Program

[a]Other races include patients of American Indian/Aleutian/Eskimo, Asian Indian, Asian Indian/Pakistani, Chamorran, Chinese, Filipino, Fiji Islander, Guamanian, Hawaiian, Hmong, Japanese, Kampuchean, Korean, Laotian, Melanesian, Micronesian, New

© 2024 Sabbagh S et al. *JAMA Network Open*

Guinean, Other, Other Asian, Pacific Islander, Polynesian, Samoan, Tahitian, Thai, Tongan, Unknown origin, and Vietnamese.
[b]Hispanic ethnicity includes patients of Mexican, Puerto Rican, Cuban, South/Central American (except Brazil), Dominican Republic, or Spanish origin.

eTable 3. Univariable Association of Factors with KRAS Testing

Characteristics	KRAS Testing		p-value
	OR	95% CI	
**Age, years**			
18 – 49	1 [Reference]	–	–
50 – 59	0.74	(0.70 – 0.80)	<0.001
60 – 69	0.59	(0.56 – 0.63)	<0.001
70 – 79	0.41	(0.38 – 0.44)	<0.001
**Sex**			
Male	1 [Reference]	–	–
Females	0.98	(0.94 – 1.02)	0.26
**Education**			
<6.3%	1 [Reference]	–	–
6.3%-10.8%	1.05	(0.86 – 1.29)	0.62
10.9%-17.5%	0.93	(0.76 – 1.14)	0.46
≥17.6%	0.77	(0.63 – 0.95)	0.01
Unknown	0.73	(0.60 – 0.90)	0.003
**Insurance status**			
Private insurance	1 [Reference]	–	–
Not insurance	0.63	(0.58 – 0.69)	<0.001
Medicaid	0.73	(0.68 – 0.78)	<0.001
Medicare/Other Governmental	0.57	(0.55 – 0.60)	<0.001
Unknown	0.60	(0.50 – 0.72)	<0.001
**Facility type**			
Academic CP[a]	1 [Reference]	–	–
Community CP[a]	1.02	(1.02 – 1.14)	0.007
Comprehensive CP[a]	0.81	(0.75 – 0.88)	<0.001
Integrated network CP[a]	0.92	(0.87 – 0.97)	0.002
**Area of Residence**			
Metro	1 [Reference]	–	–
Urban	0.91	(0.86 – 0.96)	<0.001
Rural	0.93	(0.79 – 1.09)	0.34
**Facility Location**			
New England	1 [Reference]	–	–
Middle Atlantic	0.85	(0.77 – 0.94)	0.002
South Atlantic	0.80	(0.73 – 0.88)	<0.001
East North Central	0.78	(0.70 – 0.86)	<0.001
East South Central	0.64	(0.56 – 0.72)	<0.001
West North Central	1.00	(0.89 – 1.12)	0.99
West South Central	0.72	(0.64 – 0.80)	<0.001
Mountain	0.95	(0.83 – 1.07)	0.40
Pacific	0.92	(0.83 – 1.02)	0.13
**Travel distance**			
≤5 miles	1 [Reference]	–	–
5.1-10	1.12	(1.06 – 1.18)	<0.001
10.1-25	1.16	(1.11 – 1.23)	<0.001
>25 miles	1.15	(1.09 – 1.21)	<0.001
**Year of Diagnosis**			
2010	1 [Reference]	–	–
2011	1.30	(1.18 – 1.39)	<0.001
2012	1.47	(1.36 – 1.60)	<0.001
2013	1.66	(1.53 – 1.79)	<0.001
2014	1.73	(1.59 – 1.87)	<0.001
2015	1.78	(1.64 – 1.93)	<0.001

© 2024 Sabbagh S et al. *JAMA Network Open*

Characteristics	KRAS Testing OR	95% CI	p-value
2016	1.96	(1.80 – 2.12)	<0.001
2017	1.92	(1.77 – 2.08)	<0.001
**Median Household Income**			
≥$63,333	1 [Reference]	–	–
$50,354-$63,332	0.90	(0.85 – 0.95)	<0.001
$40,227-$50,353	0.81	(0.77 – 0.85)	<0.001
<$40,277	0.72	(0.68 – 0.76)	<0.001
Unknown	1	(0.82 – 1.21)	0.96
**Charlson Comorbidity Index**			
0	1 [Reference]	–	–
1	0.90	(0.86 – 0.95)	<0.001
≥2	0.64	(0.60 – 0.69)	<0.001
**Immunotherapy**			
Not Administered	1 [Reference]	–	–
Administered	2.93	(2.78 – 3.09)	<0.001
Unknown	1.29	(0.89 – 1.87)	0.17
**Chemotherapy**			
Not administered	1 [Reference]	–	–
Administered	5.06	(4.80 – 5.33)	<0.001
Unknown	2.07	(1.82 – 2.36)	<0.001
**Surgery Primary Site**			
No	1 [Reference]	–	–
Yes	1.88	(1.81 – 1.96)	<0.001
Unknown	0.74	(0.43 – 1.27)	0.27
**Surgery Non-Primary Site**			
No	1 [Reference]	–	–
Yes	1.65	(1.57 – 1.73)	<0.001
Unknown	1.65	(0.88 – 3.10)	0.12
**Race**			
White	1 [Reference]	–	–
Black	0.89	(0.85 – 0.94)	<0.001
Others[a]	1.03	(0.93 – 1.12)	0.60
**Ethnicity**			
Non-Hispanic	1 [Reference]	–	–
Hispanic[b]	0.99	(0.91 – 1.07)	0.75

CCI: Charlson Comorbidity Index, CP: Cancer Program
[a]Other races include patients of American Indian/Aleutian/Eskimo, Asian Indian, Asian Indian/Pakistani, Chamorran, Chinese, Filipino, Fiji Islander, Guamanian, Hawaiian, Hmong, Japanese, Kampuchean, Korean, Laotian, Melanesian, Micronesian, New Guinean, Other, Other Asian, Pacific Islander, Polynesian, Samoan, Tahitian, Thai, Tongan, Unknown origin, and Vietnamese.
[b]Hispanic ethnicity includes patients of Mexican, Puerto Rican, Cuban, South/Central American (except Brazil), Dominican Republic, or Spanish origin.

© 2024 Sabbagh S et al. *JAMA Network Open*

eReferences:

1. Siegel RL, Wagle NS, Cercek A, Smith RA, Jemal A. Colorectal cancer statistics, 2023. *CA Cancer J Clin*. May-Jun 2023;73(3):233-254. doi:10.3322/caac.21772
2. Institute NC. Cancer Stat Facts: Colorectal Cancer. Accessed October 19th 2023. https://seer.cancer.gov/statfacts/html/colorect.html
3. Hong DS, Fakih MG, Strickler JH, et al. KRAS(G12C) Inhibition with Sotorasib in Advanced Solid Tumors. *N Engl J Med*. Sep 24 2020;383(13):1207-1217. doi:10.1056/NEJMoa1917239
4. Di Nicolantonio F, Vitiello PP, Marsoni S, et al. Precision oncology in metastatic colorectal cancer - from biology to medicine. *Nat Rev Clin Oncol*. Aug 2021;18(8):506-525. doi:10.1038/s41571-021-00495-z
5. Han CB, Li F, Ma JT, Zou HW. Concordant KRAS mutations in primary and metastatic colorectal cancer tissue specimens: a meta-analysis and systematic review. *Cancer Invest*. Dec 2012;30(10):741-7. doi:10.3109/07357907.2012.732159
6. Douillard JY, Siena S, Cassidy J, et al. Randomized, phase III trial of panitumumab with infusional fluorouracil, leucovorin, and oxaliplatin (FOLFOX4) versus FOLFOX4 alone as first-line treatment in patients with previously untreated metastatic colorectal cancer: the PRIME study. *J Clin Oncol*. Nov 1 2010;28(31):4697-705. doi:10.1200/jco.2009.27.4860
7. Moreira L, Balaguer F, Lindor N, et al. Identification of Lynch syndrome among patients with colorectal cancer. *Jama*. Oct 17 2012;308(15):1555-65. doi:10.1001/jama.2012.13088
8. André T, Shiu KK, Kim TW, et al. Pembrolizumab in Microsatellite-Instability-High Advanced Colorectal Cancer. *N Engl J Med*. Dec 3 2020;383(23):2207-2218. doi:10.1056/NEJMoa2017699
9. Overman MJ, McDermott R, Leach JL, et al. Nivolumab in patients with metastatic DNA mismatch repair-deficient or microsatellite instability-high colorectal cancer (CheckMate 142): an open-label, multicentre, phase 2 study. *Lancet Oncol*. Sep 2017;18(9):1182-1191. doi:10.1016/s1470-2045(17)30422-9
10. Benson AB, Venook AP, Al-Hawary MM, et al. Colon Cancer, Version 2.2021, NCCN Clinical Practice Guidelines in Oncology. *J Natl Compr Canc Netw*. Mar 2 2021;19(3):329-359. doi:10.6004/jnccn.2021.0012
11. Warren Andersen S, Blot WJ, Lipworth L, Steinwandel M, Murff HJ, Zheng W. Association of Race and Socioeconomic Status With Colorectal Cancer Screening, Colorectal Cancer Risk, and Mortality in Southern US Adults. *JAMA Network Open*. 2019;2(12):e1917995-e1917995. doi:10.1001/jamanetworkopen.2019.17995
12. Ramkumar N, Colla CH, Wang Q, O'Malley AJ, Wong SL, Brooks GA. Association of Rurality, Race and Ethnicity, and Socioeconomic Status With the Surgical Management of Colon Cancer and Postoperative Outcomes Among Medicare Beneficiaries. *JAMA Network Open*. 2022;5(8):e2229247-e2229247. doi:10.1001/jamanetworkopen.2022.29247
13. Augustus GJ, Ellis NA. Colorectal Cancer Disparity in African Americans: Risk Factors and Carcinogenic Mechanisms. *Am J Pathol*. Feb 2018;188(2):291-303. doi:10.1016/j.ajpath.2017.07.023
14. Fulton JE. Physical Inactivity is More Common among Racial and Ethnic Minorities in Most States | Blogs | CDC. Centers for Disease Control and Prevention. Accessed 10/19/2023, 2023. https://blogs.cdc.gov/healthequity/2020/04/01/physical-inactivity/
15. Gawron AJ, Yadlapati R. Disparities in Endoscopy Use for Colorectal Cancer Screening in the United States. *Digestive Diseases and Sciences*. 2014/03/01 2014;59(3):530-537. doi:10.1007/s10620-013-2937-x
16. May FP, Yang L, Corona E, Glenn BA, Bastani R. Disparities in Colorectal Cancer Screening in the United States Before and After Implementation of the Affordable Care Act. *Clin Gastroenterol Hepatol*. Jul 2020;18(8):1796-1804.e2. doi:10.1016/j.cgh.2019.09.008
17. Edwards BK, Ward E, Kohler BA, et al. Annual report to the nation on the status of cancer, 1975-

© 2024 Sabbagh S et al. *JAMA Network Open*

2006, featuring colorectal cancer trends and impact of interventions (risk factors, screening, and treatment) to reduce future rates. *Cancer*. Feb 1 2010;116(3):544-73. doi:10.1002/cncr.24760
18. Tramontano AC, Chen Y, Watson TR, Eckel A, Hur C, Kong CY. Racial/ethnic disparities in colorectal cancer treatment utilization and phase-specific costs, 2000-2014. *PLOS ONE*. 2020;15(4):e0231599. doi:10.1371/journal.pone.0231599
19. Bakkila BF, Kerekes D, Nunez-Smith M, et al. Evaluation of Racial Disparities in Quality of Care for Patients With Gastrointestinal Tract Cancer Treated With Surgery. *JAMA Network Open*. 2022;5(4):e225664-e225664. doi:10.1001/jamanetworkopen.2022.5664
20. Dharwadkar P, Greenan G, Stoffel EM, et al. Racial and Ethnic Disparities in Germline Genetic Testing of Patients With Young-Onset Colorectal Cancer. *Clin Gastroenterol Hepatol*. Feb 2022;20(2):353-361.e3. doi:10.1016/j.cgh.2020.12.025
21. Dillon J, Ademuyiwa FO, Barrett M, et al. Disparities in Genetic Testing for Heritable Solid-Tumor Malignancies. *Surg Oncol Clin N Am*. Jan 2022;31(1):109-126. doi:10.1016/j.soc.2021.08.004
22. Bilimoria KY, Stewart AK, Winchester DP, Ko CY. The National Cancer Data Base: a powerful initiative to improve cancer care in the United States. *Ann Surg Oncol*. Mar 2008;15(3):683-90. doi:10.1245/s10434-007-9747-3
23. Boehmer L, Roy UKB, Schrag J, et al. Identifying barriers to equitable biomarker testing in underserved patients with NSCLC: A mixed-methods study to inform quality improvement opportunities. *Journal of Clinical Oncology*. 2021;39(28_suppl):123-123. doi:10.1200/JCO.2020.39.28_suppl.123
24. *Assessing the Status of Biomarker Testing in Metastatic Colorectal Cancer and the Challenges Faced by Community Cancer Care Teams*. 2021. February 2021. Accessed March 20, 2024. https://www.accc-cancer.org/docs/projects/colorectal-cancer-biomarker/mcrc_survey-summary_final-(1).pdf?sfvrsn=20c6687_0
25. Petrelli NJ. A community cancer center program: getting to the next level. *J Am Coll Surg*. Mar 2010;210(3):261-70. doi:10.1016/j.jamcollsurg.2009.11.015
26. Blee SM, Shah RP, Pinheiro APM, et al. Physician Communication and Patient Understanding of Molecular Testing Terminology. *The Oncologist*. 2021;26(11):934-940. doi:10.1002/onco.13930
27. Census Bureau Data. U.S. Census Bureau. Accessed March 2024, 2024. https://data.census.gov/
28. Educational Attainment by State. World Population Review. Accessed 10/19/2023, 2023. https://worldpopulationreview.com/state-rankings/educational-attainment-by-state
29. Group. USCSW. U.S. Cancer Statistics Data Visualizations Tool. U.S. Department of Health and Human Services, Centers for Disease Control and Prevention and National Cancer Institute. Accessed March 2024, 2024. https://www.cdc.gov/cancer/dataviz
30. Umar A, Boland CR, Terdiman JP, et al. Revised Bethesda Guidelines for hereditary nonpolyposis colorectal cancer (Lynch syndrome) and microsatellite instability. *J Natl Cancer Inst*. Feb 18 2004;96(4):261-8. doi:10.1093/jnci/djh034
31. Administration USF, Drug. FDA grants accelerated approval to pembrolizumab for first tissue/site agnostic indication | FDA. Accessed 12-22, 2023. https://www.fda.gov/drugs/resources-information-approved-drugs/fda-grants-accelerated-approval-pembrolizumab-first-tissuesite-agnostic-indication
32. Aro AR, Hakonen A, Hietala M, et al. Acceptance of genetic testing in a general population: age, education and gender differences. *Patient Educ Couns*. Sep-Oct 1997;32(1-2):41-9. doi:10.1016/s0738-3991(97)00061-x
33. Sadigh G, Goeckner HG, Kazerooni EA, et al. State legislative trends related to biomarker testing. *Cancer*. Aug 1 2022;128(15):2865-2870. doi:10.1002/cncr.34271
34. Bruno DS, Hess LM, Li X, Su EW, Patel M. Disparities in Biomarker Testing and Clinical Trial Enrollment Among Patients With Lung, Breast, or Colorectal Cancers in the United States. *JCO Precis Oncol*. Jun 2022;6:e2100427. doi:10.1200/po.21.00427
35. Lenz HJ, Van Cutsem E, Luisa Limon M, et al. First-Line Nivolumab Plus Low-Dose Ipilimumab for

Microsatellite Instability-High/Mismatch Repair-Deficient Metastatic Colorectal Cancer: The Phase II CheckMate 142 Study. *J Clin Oncol*. Jan 10 2022;40(2):161-170. doi:10.1200/jco.21.01015
36. André T, Shiu K-K, Kim TW, et al. Pembrolizumab in Microsatellite-Instability–High Advanced Colorectal Cancer. *New England Journal of Medicine*. 2020;383(23):2207-2218. doi:10.1056/NEJMoa2017699
37. Zocche DM, Ramirez C, Fontao FM, Costa LD, Redal MA. Global impact of KRAS mutation patterns in FOLFOX treated metastatic colorectal cancer. *Front Genet*. 2015;6:116. doi:10.3389/fgene.2015.00116
38. Loupakis F, Ruzzo A, Cremolini C, et al. KRAS codon 61, 146 and BRAF mutations predict resistance to cetuximab plus irinotecan in KRAS codon 12 and 13 wild-type metastatic colorectal cancer. *Br J Cancer*. Aug 18 2009;101(4):715-21. doi:10.1038/sj.bjc.6605177
39. Van Cutsem E, Köhne CH, Hitre E, et al. Cetuximab and chemotherapy as initial treatment for metastatic colorectal cancer. *N Engl J Med*. Apr 2 2009;360(14):1408-17. doi:10.1056/NEJMoa0805019
40. Centers for Disease Control and Prevention (CDC). Socioeconomic factors. Accessed 12-22, 2023 https://www.cdc.gov/dhdsp/health_equity/socioeconomic.htm

© 2024 Sabbagh S et al. *JAMA Network Open*
