# Supplementary material for: Biomarker Testing Disparities in Metastatic Colorectal Cancer
Source: JAMA Netw Open. 2024 Jul 5;7(7):e2419142. doi: 10.1001/jamanetworkopen.2024.19142 (PMC11227072; doi:10.1001/jamanetworkopen.2024.19142)
Supplement: Supplement 2. — Data Sharing Statement [file jamanetwopen-e2419142-s002.pdf]

Data Sharing Statement

Sabbagh. Biomarker Testing Disparities in Metastatic Colorectal Cancer. *JAMA Netw Open*. Published July 05, 2024. doi:10.1001/jamanetworkopen.2024.19142

Data
Data available: Yes
Data types: Data dictionary, Deidentified participant data
How to access data: Data source: National Cancer Database was obtained through an application submitted by the principal investigator and coauthors. The link for the website application portal is https://www.facs.org/quality-programs/cancer-programs/national-cancer-database/puf/ Data Dictionary: Publically Available https://www.facs.org/media/brilfbgu/puf-2020-data-dictionary.pdf
When available: With publication

Supporting Documents
Document types: None

Additional Information
Who can access the data: Researchers whose proposed use of the data has been approved.
Types of analyses: For purposes related to statistical method credibility.
Mechanisms of data availability: After approval of the application submitted through the NCDB website.
